# Real-life data in 115 chronic migraine patients treated with Onabotulinumtoxin A during more than one year

**DOI:** 10.1186/s10194-016-0702-1

**Published:** 2016-12-12

**Authors:** I. Aicua-Rapun, E. Martínez-Velasco, A. Rojo, A. Hernando, M. Ruiz, A. Carreres, E. Porqueres, S. Herrero, F. Iglesias, A. L. Guerrero

**Affiliations:** 1 Neurology Department, Hospital Universitario de Burgos, Burgos, Spain; 2 Neurology Department, Hospital Clínico Universitario de Valladolid, Avda. Ramón y Cajal 3, 47005 Valladolid, Spain; 3 Neurology Department, Hospital Clínico Universitario, Valladolid, Spain

**Keywords:** Chronic Migraine, Medication overuse, OnabotulinumtoxinA, Preventatives, Real-life data

## Abstract

**Background:**

OnabotulinumtoxinA (OnabotA) is effective in Chronic Migraine (CM) during first year of treatment and longer. In real clinical setting, CM patients with acute Medication Overuse (MO) or concurrently receiving oral preventatives are treated with OnabotA. We aim to assess evolution of CM patients beyond first year on OnabotA.

**Methods:**

Data were retrospectively collected in three headache units. We analyzed cases who had received at least five sessions of OnabotA according to PREEMPT protocol. We continued OnabotA therapy when a reduction of number of headache days of at least 30% was achieved.

**Results:**

We included 115 patients (98 females, 17 males) who completed 7.6 ± 2.3 (5–13) OnabotA procedures. Previously they had not responded to topiramate and, at least, one other preventative. Age at inclusion was 45.3 ± 12 (14–74) years, and latency between CM onset and OnabotA therapy was 43.1 ± 38.2 (6–166) months. At first OnabotA session 92 patients (80%) fulfilled MO criteria and 107 (93%) received a concurrent oral preventative. In 42 cases (36.5%) OnabotA dose was increased over 155 units. After first year in 57 out of 92 patients (61.9%) MO was discontinued. Among those receiving preventatives, in 52 out of 107 they were retired (48.6%). In 22 cases (19.1%) OnabotA administration was delayed to the fourth or fifth month and in 12 (10.4%) it was temporally stopped. Finally, in 18 patients (15.7%) OnabotA was discontinued due to lack of efficacy beyond first year of treatment.

**Conclusion:**

Our results suggest that discontinuation of acute medication overuse and oral preventive therapies are achievable objectives in long-term using of OnabotA in CM patients.

## Background

Chronic migraine (CM) is an evolution of migraine, defined as headache occurring on 15 or more days per month during more than 3 months, which has the features of migraine headache on at least 8 days per month [1]. CM is estimated to affect 2% of the population [2]. Most of these patients (50–80%) fulfilled criteria of symptomatic medication overuse with all its somatic and psychological implications [2]. Besides, CM patients need preventive therapy but, excepting with topiramate, there are no controlled trials evaluating the oral preventatives commonly used in chronic migraine patients [3, 4].

After publication of the PREEMPT clinical program [5–7], in January 2012 OnabotulinumtoxinA (OnabotA) was licensed in Spain for prophylactic treatment of CM “for patients who have not adequately responded or are intolerant to prophylactic drugs for migraine”.

In a real-life setting, OnabotA can effectively reduce headache days and migraine days by at least 50%, and increase headache free days from baseline in chronic migraine sufferers [8–10]. Also in a real clinical practice, around 80% of CM patients respond to pericranial injections of OnabotA during and after the first year of therapy [11].

Our aim is to analyse real-life experience with the use of OnabotA in the treatment of patients with CM refractory or intolerant to oral preventives in three Headache Units beyond first year. We focused on symptomatic medication overuse and concurrent prophylaxis therapy.

## Methods

Cases were selected from prospective registers of patients with CM treated with OnabotA in three headache units located in three tertiary hospitals in Castilla-Leon (Spain). During the inclusion period (January 2012–January 2016), we included adult patients fulfilling criteria for CM [1]. Patients with comorbidities such as anxiety, depression or fibromyalgia and those with common vascular risk factors were also included.

OnabotA was initiated in patients who had not responded positively to at least topiramate (or another neuromodulator if topiramate was not tolerated) and a beta-blocker. We ensured that these drugs were administered at adequate doses and enough time to be effective.

Using a headache diary, patients recorded headache days, migraine days (defined as high intensity, lateralized pain with a significant impairment on daily activities) and the number of days on which they used symptomatic medication, particularly triptans, as well as the number of monthly visits to emergency department as a consequence of headache.

Exclusion criteria for the use of OnabotA were pregnant or breast-feeding women, or excessive use of alcohol. We did not exclude patients who fulfilled criteria for medication overuse, and they were allowed to continue with previous preventive oral medications with no dose increasing.

OnabotA therapy was continued when at least 30% of reduction in headache days was achieved, according to the headache diary. Patients without improvement of at least 30% reduction of headache days after three procedures were considered as no responders and OnabotA was stopped.

We retrospectively analyzed cases who had received at least five sessions of OnabotA. Statistical analysis was performed with the SPSS 20.0 statistical package.

## Results

### Demographic and baseline headache characteristics

A total of 115 patients were included, 98 females (85.2%) and 17 males. Mean age at first procedure was 45.3 ± 12 years (range 14–74 years). The latency between CM diagnosis and OnabotA therapy was 43.1 ± 38.2 months (6–166). They had received 7.6 ± 2.3 oral preventatives (range 5–13) previously to inclusion.

Among the 115 patients, 107 (93%) were receiving a concurrent oral preventive therapy when OnabotA was initiated. Besides, 92 patients (80%) fulfilled medication overuse criteria according to ICHD-3beta, including 47 (40.8%) patients overusing triptans.

In our three centers, 26 additional patients were treated with OnabotA due to a CM during the inclusion period. Among these patients, 21 did not respond after one to three procedures, one dropped out due to adverse effects, and in four cases follow-up was lost.

### Outcome

**Fig. 1:**
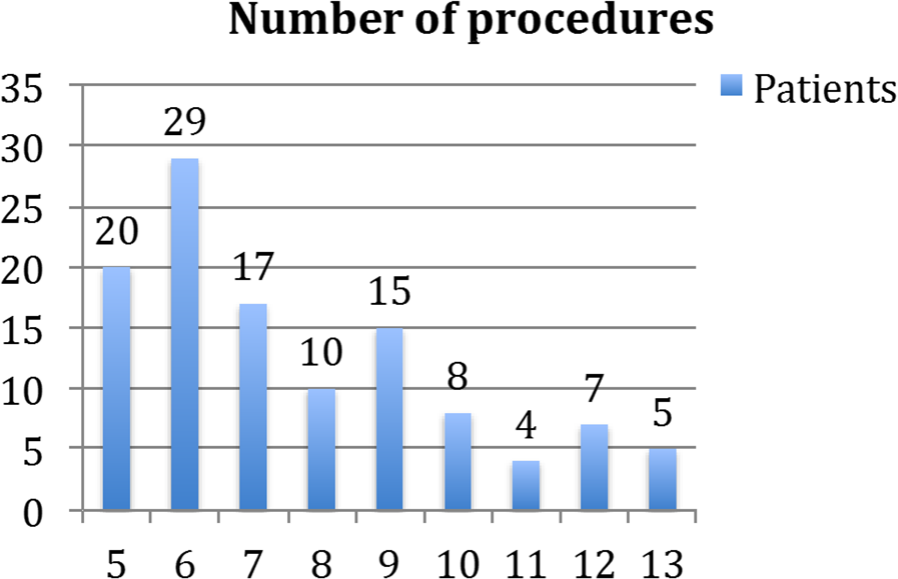
Number of OnabotA procedures in patients included in this series

Our 115 patients completed 7.6 ± 2.3 [5–13] OnabotA procedures (Fig. 1). Follow the pain protocol with additional OnabotA injections to reach up to 195 OnabotA units was used in 42 cases (36.5%), mainly when response time was shorter than 3 months.

In 79 patients (68.7%) the CM remitted to episodic migraine. When considering the concurrent prophylactic treatments, in 52 out of 115 (45.2%) cases, oral drugs were retired and in 16 (13.9%) their dose was reduced.

In 57 out of 92 (61.9%), MO was discontinued. The consumption of any kind of analgesics decreased from an average of 19.1 days per month before the first OnabotA procedure to 8.6 days per month. When considering triptans overuse, its consumption decreased from 18 to 4 days per month.

Due to a good clinical response after third procedure, we were able to delay the OnabotA session to a fourth or fifth month in 22 patients (19.1%), whilst in 12 (10.4%), it was temporally stopped.

Finally, in 18 cases (15.7%) OnabotA therapy was interrupted beyond first year due to a lack of efficacy. Most of these patients had improved between 30 and 50% during first year of treatment.

## Discussion

In our study, baseline characteristics of patients were comparable to those described in the PREEMPT trial [5–7]. Efficacy of OnabotA in our series was also similar as it was described in studies in a real-life setting [8–15].

In order to properly reflect a real-life setting, we did not exclude patients with symptomatic medication overuse. Within the PREEMPT patient population two-thirds overused acute pain medication during the 28-day baseline period [5, 6] and OnabotA was also effective in CM patients with MO [14]. Other real-life studies considered patients with medication overuse [8, 10] and OnabotA efficacy did not differ between patients with or without MO [16]. In our series, percentage of patients fulfilling medication overuse criteria was similar than observed in PREEMPT program. As additional data not previously offered in real-life studies, we achieved the discontinuation of MO in two thirds of patients.

We also included in our analysis CM patients with concurrent preventive oral therapies; in the same way that Cernuda-Morollon et al. series, percentage of patients receiving a preventative when OnabotA therapy was initiated was high [10]. One of our objectives during OnabotA treatment was to retire concurrent oral therapy and we began to decrease its use after third procedure. We were able (and this is again a point not previously considered in these kind of studies) to retire oral preventatives in almost half of our patients.

The need to modify the OnabotA injection paradigm with the “follow the pain” increasing with up to 40 additional Units remains open, and is commonly left to physician’s discretion. Negro et al. [11] showed an increased efficacy of 195 UI compared with 155 UI in a group of patients with chronic migraine with medication overuse, with no increasing in related side effects. Our study was not designed to evaluate the differences between both doses.

During last years, studies considering long-term experience with Onabot A in a real-life setting have been published [10, 17, 18]. OnabotA efficacy showed consistency after first year; percentage of patients in which OnabotA response was not maintained after first year were among 10 and 15% comparing our results with a quite similar series as that by Cernuda-Morollon et al. [10]. Another question still open to discussion is the possibility of ending OnabotA therapy after first year in patients with a good response; it has been shown that when treatment is stopped quality of life parameters worsen [17]. In the same way that previous studies [10, 18], we found that it is not easy to interrupt OnabotA therapy, even temporally. However, in nearly 20% of cases we were able to postpone OnabotA procedures to a fourth of fifth month.

## Conclusion

According to our series, OnabotA efficacy in CM patients is consistent beyond first year of treatment. Though prolonged interruption of OnabotA is difficult to achieve, we consider that discontinuation of acute medication overuse and oral preventive therapy as well as reduction of frequency of procedures are realistic goals in real-life long-term using of OnabotA in CM patients.
